# Gastric bypass surgery reveals independency of obesity and diabetes melitus type 2

**DOI:** 10.1186/s12902-016-0140-8

**Published:** 2016-11-09

**Authors:** Mogens Fenger, Dorte Lindqvist Hansen, Dorte Worm, Lisbeth Hvolris, Viggo B. Kristiansen, Elin Rebecka Carlsson, Sten Madsbad

**Affiliations:** 1 Department of Clinical Biochemistry, University Hospital of Copenhagen, Kettegaards Alle 30, 2650 Hvidovre, Denmark; 2 Department of Clinical Medicine, University Hospital of Zeeland, Køge, Denmark; 3 Department of Surgical Gastroenterology, University Hospital of Copenhagen, Kettegaards Alle 30, 2650 Hvidovre, Denmark; 4 Department of Endocrinology, University Hospital of Copenhagen, Kettegaards Alle 30, 2650 Hvidovre, Denmark

## Abstract

**Background:**

Roux-en-Y gastric bypass surgery is widely applied to ameliorate morbid obesity, including diabetes in people with type 2 diabetes. The latter vanish a few days after surgery for many, but not in all patients before any weight reduction has occurred. The explanation for this change in metabolic status is poorly understood, but the observation may suggest that the fate obesity and diabetes is only partly linked after surgery.

**Methods:**

The trajectories of weight reduction measured as reduced body mass index (BMI) in 741obese subjects with and without diabetes were evaluated. Evaluation was performed on three groups: 1) subjects that were non-diabetic before and after surgery; 2) subjects that were diabetics before surgery but non-diabetics after surgery; and 3) subjects that were diabetics before surgery and remained diabetics after surgery. The diabetic state was established at HbA1c above 48 mmol/mol.

**Results:**

The trajectories differ significantly between groups and any sub-populations of groups, the latter identified by the distance between individual trajectories using a k-means procedure. The results suggest that different domains in the enormous genetic network governing basic metabolism are perturbed in obesity and diabetes, and in fact some of the patients are affected by two distinct diseases: obesity and diabetes mellitus type 2.

**Conclusion:**

Although RYGB “normalized” many glycaemic parameters in some of the diabetic subjects apparently converting to a non-diabetics state, other diabetic subjects stay diabetic in the context of the new gut anatomy after surgery. Thus, the obesity part of the glycaemic derangement may have been ameliorated, but some defects of the diabetic state had not.

**Electronic supplementary material:**

The online version of this article (doi:10.1186/s12902-016-0140-8) contains supplementary material, which is available to authorized users.

## Background

Laparoscopic Roux-en-Y gastric bypass (RYGB) is an effective method for the treatment of patients with morbid obesity and a number of studies have shown that RYGB results in a permanent loss of up to 40 % of excess weight [1, 2]. Up to one third of the operated obese subjects present themselves with diabetes mellitus type 2 and RYGB is by far the most effective surgical procedure to obtain remission of diabetes [3] maybe except for the more radical bilio-pancreatic diversion operation [1, 3, 4]. RYGB improves glycaemic control in the diabetics already a few days after the operation and before any weight reduction has been obtained [5, 6]. The diabetic remission rate is in the range of 40–80 % in part depending on the definition of remission [1, 3, 7] and the duration of diabetes prior to surgery. Similar results have been presented for diabetic patients without severe obesity [8–10]. Thus, the primary purpose of bariatric surgery (as the name imply) was to reduce weight but has now turned to be performed in the broader context of “metabolic surgery” [2].

The main short-term mechanism behind the improvement in glycaemic control is an increase in hepatic insulin sensitivity induced by caloric restriction and an improved beta-cell function, which is unrelated to weight loss, but may be explained by the exaggerated GLP-1 response after surgery [5, 11, 12]. In contrast, improved peripheral (skeletal muscle) insulin sensitivity is strongly associated with weight loss [12–14]. However, the relative importance of caloric restriction versus the changes in gut hormone secretion for the remission of diabetes is still discussed [15, 16]. GLP-1 has strong insulinotropic effects and inhibits glucagon release and, in complex interactions with glucose-dependent insulinotropic polypeptide (GIP), defines the much favoured lower intestine hypothesis of diabetic remission. The ghrelin hypothesis states that ghrelin secretion from the stomach and proximal intestine is disturbed decreasing appetite and fat mass, while the upper intestine (anti-incretin) hypothesis suggests that some unknown factors or processes in the duodenum influencing the glucose homoeostasis and impaired in type2 diabetes are altered by RYGB [2, 17]. These hypotheses including the effect of calorie restriction may not be exclusive, but for now the exact physiology behind the remission of diabetes in some but not all patients remains elusive.

In the present contribution we evaluated the trajectories of weight reduction measured as reduction in body mass index (BMI) applying information theoretic approaches to RYGB-treated obese patients with and without diabetes. Although the mechanism(s) of the vanishing of diabetes is far from resolved our results suggest that different domains of the genetics network governing the basic metabolic processes leading to obesity and diabetes are perturbed in these heterogeneous conditions. Obesity is considered a risk factor for developing diabetes, but the link may be quite vague, as only 30 % of our morbid obese patients do present themselves with diabetes. In addition both conditions are polygenic in nature and are therefore heterogeneous entities considering the vast number of genetic mutations detected in human genome [18].

## Methods

### Population

The subjects included in the study have all been treated by gastric bypass surgery for severe obesity (bariatric surgery). A total population of 1189 subjects, all of Caucasian ethnicity, have been described and monitored clinically and para-clinically from June 2009 to November 2014. Surgery was performed using a standard laparoscopic RYGB technique creating a 25 ml gastric pouch a 75 cm long bilio-pancreatic limb and a 100 cm long Roux limb as previously described [19].

Blood, serum, plasma and urine were obtained at each (pre- and postsurgical) visit to the clinic and amounts to more than 2500 serum/plasma and urine sample. Full records are available for 741 patients in whom laparoscopic “Roux-en-Y gastric bypass” (RYGB) were performed and forms the core in this contribution. The follow-up time after surgery was 22.6 month on average (range 3–77 months).

The population was divided in three sub-populations before surgery: 1) a sub-population which were non-diabetic before and after surgery; 2) a diabetic sub-population defined purely by the relative concentration of glycated haemoglobin (HbA1c) according to the IFCC standard (HbA1c >48 mmol/mol) at any time (glycaemic diabetics); and 3) a diabetic sub-population with HbA1c below the cut-off levels at all time, but treated with anti-diabetic agents (treatment diabetics). The rational for this distinction between diabetic patients, is that patients with HbA1c above the cut-off level regardless of treatment genuinely are diabetics by definition. Treated patients (anti-diabetic drugs or insulin) with levels below the cut-off at all times may have been diabetics before surgery but turned non-diabetic due to weight loss but were still treated with anti-diabetic drugs. The glycaemic diabetic sub-population was further divided into two subgroups, one that converted to non-diabetics post-surgery without any anti-diabetic treatment, and a subgroup receiving anti-diabetic agents.

The glycaemic status was not determined in 6 RYGB-patients who were excluded from further analysis.

### Biochemical analysis

The HbA1c level was determined by liquid chromatography (Tosoh, Alere, Belgium) in a standard setup used for routine clinical diagnostics. Haemoglobin and blood cell counts were performed on SysMex 500 (Sysmex, Japan) using standard protocols. INR analysis and calculation were performed by ACL TOP (IL, Italy). All other variables were analysed on Cobas 6000 (Roche Hitashi, Japan). All analyses were performed using the standard protocols as recommended by the manufactures used for routine clinical diagnostics. All analyses were performed the day of sampling.

The homoeostases model assessment [20] was used to estimate insulin resistance (HOMA-IR) and pancreatic β-cell function (HOMA-beta).

### Basic data analysis

Relative changes in variables were calculated as the minimal post-surgery value compared to the latest pre-surgery value. If a variable increased post-surgery the changes are shown as negative values. Variables for which no changes after surgery were observed were excluded from further analysis as they were non-informative for the physiological processes related to obesity and diabetes. The pre- and post-surgery values as well as the relative changes were pooled in each sub-population and for both gender separately for all traits. The cumulative distributions of the traits were compared pair-wise by the Kolmogorov-Smirnov two-sample test as implemented in http://scipy.org/. This is a non-parametric test of two empirical samples of continuous variables defining the largest absolute difference between the two cumulative distributions. The test is symmetric, do not require equal sample size and generates a *p*-value for significance. Increasing test-statistics (and hence decreasing *p*-values) indicate increasing dissimilarity of distributions and hence weaker correlation. The idea of these analyses is that traits should have similar distributions (of any kind) to be correlated, and the more similar they are the closer the interactions of the traits.

### Classification of sub-populations

The sub-populations are supposedly heterogeneous [18], and were disentangled into classes using a k-means procedure. For the purpose of analysis of the structure of the population the data from each gender were pooled as in the vast majority of traits no gender difference were detected (Additional file 1: Table S1). The time-series data used for classification were normalized BMI (normalized to the pre-surgery value).

The time series for each subject in each sub-population were fitted to polynomials of increasing degree using the polyfit and polyval functions in numpy (http://scipy.org/). The polynomial degree with the lowest Akaike’s information criterion (AIC) value or minimum residuals were selected. It should be noted that subjects were only included if the number time-points equals or are larger than the degree of the polynomials. Therefore, with increasing degree of polynomials, the size of the sub-population shrinks so not all polynomials (subjects) were classified. However, a crude estimation of the structure of the sub-populations were obtained.

The classification in two steps: 1) The Kullback-Leibler divergence or relative entropy [21] were calculated for all pairs of polynomials (equal to the number subjects in the sub-populations conditional on the degree of the polynomials above) with each subject in turn representing the true distribution. The outcome is a n*n matrix holding all the entropy values from the comparison of the polynomial. The entropy is a measure of information in the data sets such that the closer the polynomials are the smaller is the entropy i.e. the smaller is the loss of information. 2) The matrices (one for each clinical condition) were used in k-means classification procedures (cluster.vq procedure in scipy (http://scipy.org/); KMeans in sklearn.cluster from scikit (http://scikit-learn.org/)) with increasing number of classes. The k-means works by randomly selecting one or more polynomials (subjects) in the entropy-list as centroids and then clustering the polynomials with the lowest divergence with or distance to the centroid. This were done with 2500 seeds of centroids in each classification procedure and a convergence threshold of 1*E-5 to avoid local minima in the scipy k-means procedure. In the scikit procedure 100,000 seeds were used. The iteration stopped when one of the two criteria (threshold or number of seeds) was reached first. The output is a code-book (list of polynomials) belonging to the same cluster or class for the best fit. This procedure were implemented for increasing number of clusters (classes) and for goodness-of-fit the Akaike information criteria AIC were used to decide on the number of clusters (classes) in the sub-population. The difference between successive classes AIC is chi-square distributed and when the associated *p*-value was higher than the decreasing Bonferoni *p*-value (number of classes) the classification procedure were stopped. The penultimate classification was then defined as the optimal classification.

It is presumed and intended that the procedure generates homogeneous populations (which of course is an over-simplification) and the time-series where therefore collated in one composite time-series for each class. The classes where then evaluated within a sub-population (to confirm the separation of classes) and between sub-populations (diabetics and non-diabetics) using the Kolmogorov-Smirnov two-sample test as described above.

### Statistics

All statistics were done using the numpy and scipy packages available from SciPy.org on the Ubuntu 14.04 LTS Linux platform coded in Python using the Spyder 2.2.5 interpreter under the terms of the MIT License. Power calculations were done using the power_t_test algorithm from The Comprehensive R Archive Network (CRAN, The R Foundation for Statistical Computing) using the rpy2 Python interface. All the software are open source and freely available.

## Results

### General description of the data

The basic data for the included patients is shown in Table 1. The majority of the patients were females (71.5 %), but sex distribution differs significantly between non- diabetics and diabetics, where the two genders were equally represented in the last group. Thirty percent of the patients were classified as diabetics, when both categories of diabetics were included. The age at surgery was significantly lower in the non-diabetic group (NDM) group compare to the two diabetic groups.

**Table 1:** Clinical status. Age at surgery and time to post-surgery non-diabetic state for pre-surgery diabetic subjects

Diabetics (DM)	223								
*By glycemic status*	100								
NDM post surgery	48								
DMT2 post surgery	34								
NDM post/treat DMT2	17	(see text)							
*By treatment only*	123								
NDM post surgery	112								
DMT2 post surgery	11								
Non-diabetics (NDM)	518								
Not determined glycemic	5								
Not determined glycemic/treat DMT2	1								
Total	747								
Fraction diabetics	0.3								
Females	530								
Males	217								
Age at surgery	Glycemic DMT2			Treatment DMT2			NDM		
	Females	Males	Females %	Females	Males	Females %	Females	Males	Females %
Number	54	46	54.0 %	70	53	56.9 %	402	116	77.6 %
Mean	49.58	50.31		48.55	48.5		41.97	41.02	
Minimum	30.79	30.29		27.08	27.49		18.97	19.05	
Maximum	64.65	65.53		61.95	61.83		65.37	59.18	
Variance	71.66	50.14		77.68	69.14		91.38	83.39	
Std	8.47	7.08		8.81	8.31		9.56	9.13	
Females-Males. *p*-value*		0.642			0.975			0.33	
Compare clinical status	Females	Males							
NDM-Glycemic	<0.001	<0.001							
NDM-Treatment	<0.001	<0.001							
Glycemic-Treatment	0.512	0.248							
Days to NDM after surgery	Glycemic DMT2			Treatment DMT2					
	Females	Males		Females	Males				
Number	33	32		59	41				
Mean	202.55	201.94		146.64	178.61				
Minimum	42	84		11	66				
Maximum	708	696		451	751				
Variance	21040	18975		8088	23356				
Std	145	137		90	153				
Females-Males. *p*-value		0.986			0.239				

Glycemic DMT2 refers to patients classified as diabetics if HbA1c exceeds 48 mmol/mol regardless of any treatment. The Treatment DMT2 sub-population includes all subjects with HbA1c below 48 mmol/mol but received antidiabetic medical treatment (drugs or insulin)

*The *p*-values are given as nominal values. Only *p*-values <0.001 can be considered significant after Bonferoni correction for multiple testing

Almost half of the glycaemic diabetics, DMT2, turned non-diabetics after surgery, NDM, (DMT2-NDM sub-population) to which may be added the 17 patients receiving post-surgery (but not pre-surgery) anti-diabetic treatment. This number may be even higher as the 34 patients still diabetics (DMT2-DMT2) according to the HbA1c levels may have shorter follow-up times and with time and weight loss may actually convert to a non-diabetic state. The average time for conversion in this group was 202 days (range 42 to 708 days) as judged from the HbA1c levels. Notably, this observation may, at least in part, be explained by the half-life of HbA1c and time span between outpatient visits, since weight loss is ongoing the first 1–2 years after RYGB. The fate of the treatment DM group differs from the glycaemic DM group: the time to NDM-conversion was shorter and more than 91 % did convert to NDM post-surgery, while only 65 % converted in the glycaemic DM group (including the 17 subjects prescribed anti-diabetic treatment post-surgery, Table 1). The post-surgery NDM status in this group was defined as HbA1cIFCC values below 48 *and* medical treatment withdrawn. There were no differences between genders within each sub-population.

The actual diabetic status of the treatment DM group was not unambiguously defined and for some variables this group was similar to the glycaemic DM groups, in others to the NDM group (see also Additional file 1: Table S1). Therefore the treatment DM group was not further included in interpretation of the RYGB data.

All data for all the variables in each sub-population are listed in Additional file 1: Table S1. An excerpt of the primary outcome variables (HbA1c, insulin and glucose) is tabulated in Table 2 (see also Additional file 2: Table S2). The relative changes (pre- to post-surgery) were significant for weight, BMI and the HbA1c measures in all populations (NDM, DMT2-NDM and DMT2-DMT2). The average weight loss and decrease in BMI were approximately 25 %, but varies considerably from almost no decrease up to more than 50 %, suggesting very heterogeneous sub-populations of weight loss responders. The changes in the NDM and the DMT2-NDM sub-populations were marginal significantly different for weight and BMI for females but not for males.

**Table 2:** Maximal changes in glucose-related variables

HBA1CIFCC (mmol/mol) Range <48						
	NDM		DMT2-NDM		DMT2-DMT2	
	Pre-surgery	Pre-surgery	Pre-surgery	Post-surgery	Pre-surgery	Post-surgery
Number of subjects	179		47		8	
Mean	37	32	63	38	84	53
Range	27–47	24–43	42–113	28–46	54–122	48–70
*Comparison of conditions,*	p-values*					
	Pre-surgery	Post-surgery				
NDM v DMT2-NDM	<0.001	<0.001				
NDM v DMT2-DMT2	<0.001	<0.001				
DMT2-NDM v DMT2-DMT2	0.031	<0.001				
Glucose (mmol/L) Range 4.2–6.1						
	NDM		DMT2-NDM		DMT2-DMT2	
	Pre-surgery	Post-surgery	Pre-surgery	Post-surgery	Pre-surgery	Post-surgery
Number of subjects	182		43		7	
Mean	5.4	4.9	8.8	5.8	9.8	6.4
Range	3.9–8.0	1.3–7.1	3.3–24	4.1–9.1	3.4–19	4.8–8.4
*Comparison of conditions,*	*p-values*					
	Pre-surgery	Post-surgery				
NDM v DMT2-NDM	<0.001	<0.001				
NDM v DMT2-DMT2	0.070	0.032				
DMT2-NDM v DMT2-DMT2	0.647	0.323				
Insulin (pmol/L) Range 10–125						
	NDM		DMT2-NDM		DMT2-DMT2	
	Pre-surgery	Post-surgery	Pre-surgery	Post-surgery	Pre-surgery	Post-surgery
Number of subjects	177		42		7	
Mean	113	41	150	50	69	32
Range	23–486	1–112	13–494	2–11	4–167	1–53
*Comparison of conditions,*	*p-values*					
	Pre-surgery	Post-surgery				
NDM v DMT2-NDM	0.020	0.019				
NDM v DMT2-DMT2	0.074	0.176				
DMT2-NDM v DMT2-DMT2	0.007	0.022				
HOMA-IR						
	NDM		DMT2-NDM		DMT2-DMT2	
	Pre-surgery	Post-surgery	Pre-surgery	Post-surgery	Pre-surgery	Post-surgery
Number of subjects	175		42		7	
Mean	28.1	9.5	59.1	13.5	36.8	9.3
Range	4.7–164.0	0.3–28.7	1.9–199.0	0.6–36.7	0.6–107.6	0.3–15.4
*Comparison of conditions,*	*p-values*					
	Pre-surgery	Post-surgery				
NDM v DMT2-NDM	<0.001	<0.001				
NDM v DMT2-DMT2	0.564	0.925				
DMT2-NDM v DMT2-DMT2	0.190	0.082				
HOMA-BETA						
	NDM		DMT2-NDM		DMT2-DMT2	
	Pre-surgery	Post-surgery	Pre-surgery	Post-surgery	Pre-surgery	Post-surgery
Number of subjects	174		41		6	
Mean	1303	489	869	430	239	261
Range	236–5215	40–2072	113–1101	8–1184	88–369	121–546
*Comparison of conditions,*	*p-values*					
	Pre-surgery	Post-surgery				
NDM v DMT2-NDM	0.023	0.204				
NDM v DMT2-DMT2	<0.001	0.020				
DMT2-NDM v DMT2-DMT2	0.001	0.063				

Subjects were only included if both pre- and post-surgery values were available

NDM, glycemic non-diabetics pre- and post-surgery

DMT2-NDM; pre-surgery glycemic diabetics, post-surgery glycemic non-diabetics

*The *p*-values are given as nominal values. Only *p*-values <0.001 can be considered significant after Bonferoni correction for multiple testing

The relative changes in HbA1c were significant except for the DMT2-DMT2 females. Pre- to post-surgery changes in HbA1c values differed significantly between the NDM and DMT2-NDM sub-populations, and to a lesser extent between the NDM and DMT2-DMT2, although the values were highly variable. The difference of post-surgery levels NDM and DMT2-NDM sub-populations compared to DMT2-DMT2 is of course due to the classification criteria (see Methods). The relative changes were 2.6 to 3.8 fold higher in the diabetic sub-populations than in the non-diabetic sub-population, but nevertheless the change in the NDM group was significant and the metabolic state was “improved” in this otherwise non-diabetic sub-population. The post-surgery levels of HbA1c in both the diabetic groups approached pre-surgery but not the post-surgery levels in the non-diabetics.

Insulin decreased 45–60 % post-surgery irrespective of the pre-surgery levels and glucose tolerance, except for females in the DMT2-DMT2 sub-population (Additional file 2: Table S2). There were no significant differences of the relative changes in level between the sub-populations and all post-surgery levels were within the normal range. However, the pre-surgery levels showed a complex pattern: the mean levels were increased in DMT2-NDM sub-population and the male NMD sub-population, but not in in the female NDM sub-population and in the DMT2-DMT2 sub-populations (Table 2 and Additional file 2: Table S2). Also, some subjects in the NDM sub-populations had increased pre-surgery insulin levels, suggesting that the metabolic steady state in the non-diabetic subjects (as judge by the HbA1c levels) were heterogeneous i.e. may be defined as pre-diabetics.

A similar pattern was observed for fasting glucose levels with significant differences between pre- and post-surgery levels and relative changes (8 % in the non-diabetic sub-population up to 26 % in the DMT2-NDM sub-populations, Additional file 2: Table S2). On average, glucose levels were normalized in the NDM and DMT2-NDM sub-populations, but not in the DMT2-DMT2 sub-population and some subjects did have persistent hyperglycemia post-surgery in all sub-populations. Importantly, the post-surgery level in diabetics did not reach the pre-surgery level of the non-diabetics and not at all the non-diabetic post-surgery level for at least some of the subjects in the diabetic sub-populations.

The HOMA-beta and HOMA-IR indices decreased 53–60 % in the NDM sub-populations irrespective of gender (Table 2). No significant changes were seen in the diabetic sub-populations except for HOMA-IR in the male DMT2-NDM sub-population (Additional file 3: Table S3). The trends were however similar for HOMA-IR in all sub-populations with a substantial increase in insulin sensitivity. Importantly, the pre- and post-surgery levels of HOMA-IR were significantly different comparing NDM and DMT2-NDM (Table 2). The NDM and DMT2-NDM sub-population had similar levels of HOMA-beta values and seemed distinctively different from the DMT2-DMT2 sub-population, but these differences did not reach statistical significance. It should be noted that the number of subjects in the DMT2-DMT2 sub-population was low and variances rather high.

C-peptide decreased 34–44 % post-surgery with no difference in the NDM and DMT2-NDM sub-populations. The decreases in post-surgery levels in the DMT2-DMT2 sub-population were notable, but did not reach significance. The C-peptide levels were only marginal different when the NDM and DMT2-NDM subpopulations were compared (Additional file 2: Tables S2).

The changes in lipids were rather complex. Cholesterol decreased 17–26 % after surgery in the NDM and female DMT2-NDM sub-populations, and although substantial decreases were also seen in the DMT2-DMT2 sub-populations they did not reach significance. A similar pattern was seen for LDL-cholesterol. Triglyceride and VLDL-cholesterol levels decreased substantially in all sub-populations although the decreases were only marginal in the DMT2-DMT2 sub-populations. Small increases of HDL-cholesterol were detected. HDL-cholesterol was within the normal range except for a few subjects deviating slightly from the normal range.

The low-grade inflammatory parameter C-reactive protein decreased to a large extend in all sub-populations and gender (56–79 %), although the changes did not reach significance in the male DMT2-DMT2 sub-population. The decrease was much less for leukocytes (14–25 %) but still significant except for DMT2-DMT2 sub-populations. With a few exceptions all subjects were within the normal range for C-reactive protein and leukocytes.

A summary of the relative changes for all the variables is shown in Additional file 3: Table S3. The significance of the relative changes were high (*p* <0.001) for most of the variables in the non-diabetic sub-population particular for women and with high power (>0.95). A similar pattern was seen for the DMT2-NDM sub-population, but important differences were observed. The most striking observation was the high responsiveness of the NDM sub-population to RYGB where many variables were “normalized” compared to the DMT2-NDM sub-populations and in particularly to the DMT2-DMT2 sub-populations in which only minor changes occurred (the number of subjects is small and variances may be large reducing the reliability of the statistics).

### Trait distributions

The Kolmogorov-Smirnov analyses of similarity of trait distributions are very complex. If a level of acceptance of similarity is set at 0.05 then 311 similarities were observed among all the patients. However, such a level translates into low correlation. The more stringent level of 0.9 reduced the number of similarity of two-trait distributions to 55 (44 unique) as shown in Additional file 4: Table S4. Only the weight-BMI association was observed for all sub-populations. Taken all together, most of the associations were consistent with the concept of the metabolic syndrome including insulin, C-peptide, lipids and inflammatory markers.

The comparison of trait distributions within gender between the NDM and DMT2-NDM sub-populations are shown in Additional file 5: Table S5. The similarity *p*-values were significant for all traits in the female population, but 13 traits in the male populations were not significant. Except for the HOMA indices and TSH significant differences were observed for in at least one of the genders. Thus, the vast majority of trait distributions do not emerge from a common distribution, suggesting basic differences in metabolic physiology in non-diabetics and diabetics even if the latter turned non-diabetic after surgery.

### Classification of sub-populations

**Fig. 1:**
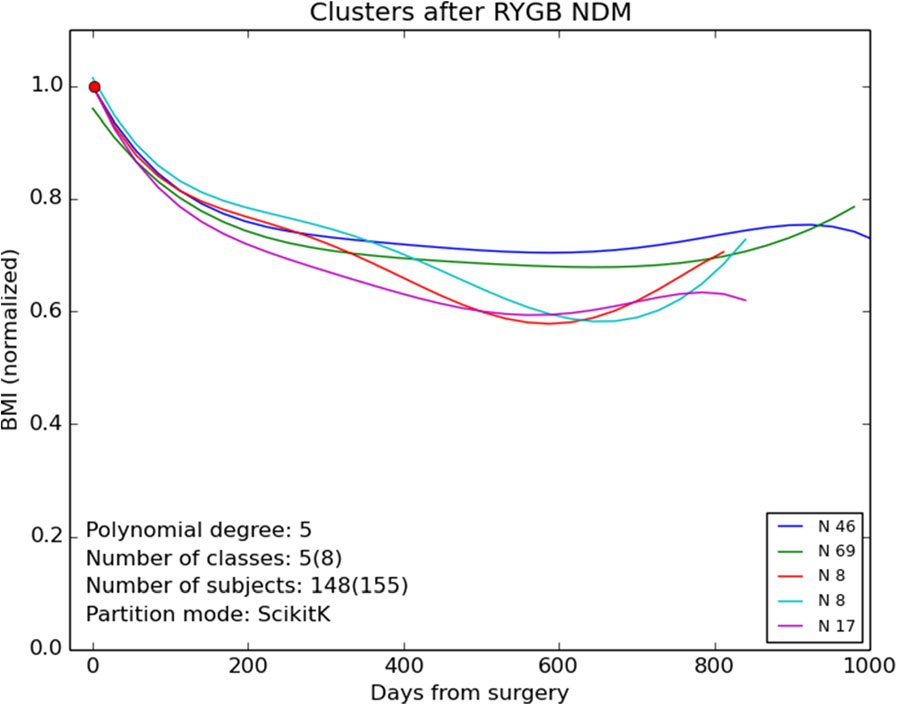
Trajectories of the five clusters (out of 8) containing five or more subjects. The data was pooled for all the subjects in the clusters increasing the number data points to no less than 40, i.e. 148 subjects and not less than 750 measurements were included in the analysis. Here the trajectories for the non-diabetic sub-populations are shown. The *red dot* indicates the point of normalization of BMI at time zero i.e. the penultimate data points before surgery. Although the trajectories seem alike most of them differed significantly. See Additional file 6: Table S6 for statistical data for the clusters for all sub-populations (including the diabetic sub-populations)

The polynomial degree was 5 for non-diabetics and 4 for the diabetic sub-populations (not shown). The classification procedures detected five classes in an 8-class procedure in non-diabetics (NDM) where classes with less than 5 polynomials (subjects) were ignored (Additional file 6: Table S6). Eight of the ten comparisons were significant. In all four classes may be discerned. Only two different classes were detected in both the diabetic sub-populations. The time courses of the classes for the non-diabetics are plotted in Fig. 1.

In Table 3 the comparison of distributions between diabetics and non-diabetics are summarized. The analysis was restricted to classes with 5 or more subjects. As the comparison of the NDM classes 3 and 4 did not differ and the comparison with the DMT2-DMT2 subpopultions also did not differ significantly the number of distinctive classes in the entire population are 11.

**Table 3:** Comparison of clinical conditions

NDM v DMT2-NDM				
		DMT2-NDM		
Class number		1	2	Total
	Subjects	6	12	18
NDM				
1	46	<0.01*	<0.01	
2	69	<0.01	<0.01	
3	8	0.03	0,25	
4	8	0.09	0.10	
5	17	<0.01	0.02	
Total	148			
NDM v DMT2-DMT2				
		DMT2-DMT2		
Class number		1	2	Total
	Subjects	17	7	24
NDM				
1	46	<0.01	<0.01	
2	69	<0.01	<0.01	
3	8	<0.01	0,95	
4	8	<0.01	1.00	
5	17	0.93	<0.01	
Total	148			
DMT2-NDM v DMT2-DMT2				
		DMT2-DMT2		
Class number		1	2	Total
	Subjects	17	7	24
DMT2-NDM				
1	6	<0.01	0.21	
2	12	0.21	0.08	
Total	18			

The clusters or classes should contain at least 5 subjects to be included in the analysis

NDM, glycemic non-diabetics pre- and post-surgery

DMT2-NDM; pre-surgery glycemic diabetics, post-surgery glycemic non-diabetics

DMT2-DMT2; pre-surgery glycemic diabetics, post-surgery glycemic diabetics

*The *p*-values are given as nominal values. Only *p*-values <0.01 can be considered significant after Bonferoni correction for multiple testing

## Discussion

RYGB surgery is superior to medical treatment in optimizing glycemic control [22] and claimed in many studies to resolve diabetes mellitus in most obese with diabetes. This is, however, not exactly true as RYGB defines a new metabolic and physiological framework (see also [23]). First, HbA1c and glucose levels in the diabetics did just barely reach the pre-surgery levels in the non-diabetic, but certainly did not approach the post-surgery levels of the non-diabetics. This is particularly the case for the diabetic sub-population that apparently turned non-diabetic after surgery, i.e. they did not become true non-diabetics. Second, the distributions of the traits differed markedly between and within sub-populations. Third, at least 11 classes or sub-populations are present in the entire study population each representing different physiological statuses. This is particularly apparent when the NDM and the DMT2-DMT2 sub-populations were evaluated, but importantly also when NDM and the DMT2-NDM sub-populations were compared. Although several variables were or became normalized after surgery the control population is not the usual non-treated matches but rather the non-diabetic RYGB population, and hence a new physiological set-point is defined. Thus, the diabetics stayed diabetics. It should be noted that even in the non-diabetic sub-population gross deviations of e.g. insulin is seen, that is the non-diabetic sub-population may in fact not be that non-diabetic.

The actual important physiological changes resulting after RYGB or other bariatric procedures are unclear [15, 24]. Lack of a universal definition of remission of diabetes [3], different study designs and small populations have hampered our understanding of the RYGB influence on the glycaemic state. An important question is whether weight-independent effects on the glycaemic state are present. Some studies ascribe the glycaemic improvement to the weight loss [12, 14], but another study showed that weight gain that occurred in ~1/3 of the diabetics two years after RYGB did not influence the improvement in peripheral or hepatic insulin sensitivity [25]. In addition, the improvement of glycaemic status shortly after surgery without weight loss has been shown in several studies [8], although the effect of calorie restrictions cannot be ruled out in these studies [12, 13]. On the other hand it is widely accepted that peripheral improvement of insulin sensitivity requires a moderate to substantial weight loss [13, 14] at least to settle the improvement, as later weight gain 1–2 years post-surgery did not deteriorate the metabolic improvement obtained [21].

The metabolic blueprint is residing in the genome as complex networks of interacting genes and regulatory structures [18, 26]. Myriads of polymorphisms and mutations have been detected, not the least due to the explosion of massive sequencing. Thus, variations in genetic structures translates into variations of genetic networks and hence the expressed phenotypes. The 11sub-populations detected in the present study only represent a tiny fraction of the unknown real number of (homogeneous) sub-populations. The simple procedures here applied to time-series data are insufficient to define exact phenotypes. Structural equation modelling combined with latent profiling and transfer entropy analysis will provide an avenue to refinement in phenotype analysis and definition [18, 27, 28]. The precise definition of the phenotypes is at the heart of a subsequent genetic analysis, if such an analysis should have any hope of defining just the important parts of the networks governing the metabolism.

## Conclusions

Gastric bypass (RYGB) defines a new metabolic and physiological framework that as a consequence requires a redefinition of the diabetic status after gastric bypass surgery. Hence, adapting the non-diabetics glycemic status post-surgery as a reference the diabetics stayed diabetic although some (but not all) diabetics would be classified as non-diabetics according to the usual reference values and levels. The significance of this is not clear and is complicated by the heterogeneity of the population. This heterogeneity can be envisioned as a continuous distribution of physiological states determined by genetic and environmental factors [18], which in turn will determine the consequences if any of the new metabolic state of the obese subjects.

## Additional files

Additional file 1: Table S1.
*Maximal changes in variables and the day after RYGB.* Values and summary statistics of all variables analysed before and after gastric bypass for the subpopulations non-diabetics, diabetics turned non-diabetics post-surgery and diabetics that stayed diabetics post-surgery. Values and statistics are given for female and males, respectively. (XLS 226 kb)
Additional file 2: Table S2.
*Maximal changes in glucose-related variables.* Values and summary statistics for glucose-related variables and diabetes for both gender combined. An excerpt of the table of the central variables related to diabetes is presented in Table 2. (XLS 48 kb)
Additional file 3: Table S3.
*Relative changes in variables after RYGB.* The changes in all traits are calculated as fraction relative to pre-surgery values for non-diabetics and diabetics turned non-diabetics, respectively. The changes and significance are calculated for female and males separately. (XLS 28 kb)
Additional file 4: Table S4.
*Komogorov-Smirnoff test for equality of distributions of traits.* Traits are pair-wise tested for distributional similarity of the time-evolving changes in trait values (trajectories), that is to which extent the traits may be correlated. The comparisons of distributions are done for all subpopulations and gender separately. (XLS 22 kb)
Additional file 5: Table S5.
*Komogorov-Smirnoff test for equality of distributions of traits between NDM and DMT2-NDM.* Traits are tested for distributional similarity between the non-diabetic subpopulation and the diabetic turned non-diabetic subpopulation. (XLS 22 kb)
Additional file 6: Table S6.
*Clustering summary using the scikit k-means procedure.* The three subpopulations non-diabetics, diabetics turned non-diabetics and diabetics staying diabetics after gastric bypass were partitioned into subpopulations using the k-mean procedure for changes in body mass index (BMI) during time. In all, at least 11 subpopulations were identified. (XLS 21 kb)

## Abbreviations

BMI: Body mass index
DMT2: Diabetes mellitus type 2 defined by HbA1c
DMT2-DMT2: Pre- and postsurgery diabetes mellitus type2
DMT2-NDM: Pre-surgery diabetes mellitus type2 turned non-diabetics post-surgery
GIP: Glucose-dependent insulinotropic polypeptide
GLP-1: Glucagon-like peptide 1
HDL: High density lipoprotein
HOMA-beta: The homeostases model assessment to pancreatic β-cell function
HOMA-IR: The homeostases model assessment to estimate insulin resistance
INR: International normalized ratio of prothrombin time
LDL: Low density lipoprotein
NDM: Non-diabetics
RYGB: Laparoscopic Roux-en-Y gastric bypass
VLDL: Very low density lipoprotein
